# Liquefaction analysis of marine diversion dike foundation and gravel pile reinforcement treatment based on the PL-Finn model

**DOI:** 10.1371/journal.pone.0330325

**Published:** 2025-08-12

**Authors:** Jie Zhao, Yahui Lin, Yijiang Fan, Xiaodong Yu

**Affiliations:** 1 School of Architectural Engineering, Dalian University, Dalian, Liaoning, China; 2 Dalian Public Transportation Construction & Investment Group Co., Ltd., Dalian, Liaoning, China; Maria Curie-Sklodowska University: Uniwersytet Marii Curie-Sklodowskiej, POLAND

## Abstract

This study investigates the liquefaction characteristics and failure mechanisms of sand foundations in the marine diversion dike of a coastal nuclear power plant, along with the anti-liquefaction performance of gravel pile-reinforced foundations. Using the three-dimensional finite difference software FLAC^3D^ (Fast Lagrangian Analysis of Continua), we conducted numerical simulations of both unreinforced and reinforced liquefiable sand foundations. A three-dimensional model was developed, incorporating dynamic analysis with the PL-Finn constitutive model to simulate post-liquefaction large deformations. The efficacy of compacted gravel piles was evaluated through residual deformation, excess pore water pressure, and pore pressure ratio. Results demonstrate that unreinforced foundations exhibit systematic residual deformation due to liquefaction-induced sand flow, which is significantly reduced by gravel pile reinforcement. Both excess pore water pressure and pore pressure ratio decrease markedly after reinforcement. This substantially enhances the composite foundation’s structural stability and liquefaction resistance. These findings confirm that compacted gravel pile groups provide effective drainage and anti-liquefaction capacity, meeting site liquefaction mitigation requirements under design-level ground motion intensity, thereby offering a reference for similar engineering projects.

## 1 Introduction

Historical seismic damage records indicate that structural failures in diversion dikes are frequently caused by foundation sand liquefaction. Consequently, foundation treatment for diversion dike structures constitutes a critical aspect of earthquake and liquefaction resistance. For many years, researchers worldwide have focused on enhancing the liquefaction resistance of diversion dikes, with the goal of mitigating structural damage and losses during earthquakes. Numerous scholars have conducted extensive model testing and numerical analysis to investigate the seismic performance of these structures, yielding significant findings. Yang et al. [1] employed LS-DYNA software to perform dynamic elastic-plastic analysis, incorporating soil-structure interaction (SSI). They analyzed the seismic response of a nuclear power plant seawall and the influence of seismic wave parameters, providing theoretical support for its design and seismic performance. Liu et al. [2] studied the seismic response of the diversion dike head at a coastal nuclear power plant. They established a three-dimensional model using ANSYS and employed FLAC^3D^ coupled with the PL-Finn model for static and dynamic analysis. Zhao et al. [3] utilized the Hardin-Drnevich equivalent linear model to establish a two-dimensional model of a nuclear power plant diversion dike. Using a dynamic effective stress analysis method, they calculated the stability safety factor, residual deformation, and liquefaction area under SL1 and SL2 earthquake conditions.

Research on soil-structure dynamic interaction (SSI) originated from the study by Reissner et al. [4] on vibrations of rigid circular foundations on an elastic half-space surface. With advances in modern numerical methods and computer technology, as well as the successive construction of major engineering projects, SSI research under seismic loads has progressed significantly in both theory and practice, yielding substantial advances [5–9].

By reinforcing liquefiable soil layers with gravel piles to form a composite foundation, the bearing capacity is enhanced, settlement deformation reduced, and excess pore water pressure dissipation accelerated due to high permeability [10]. Gravel piles are extensively adopted in engineering practice owing to readily available materials, rapid construction, and cost-effectiveness [11]. Despite these applications, theoretical understanding—particularly regarding seismic liquefaction resistance—lags behind. Current findings primarily rely on field tests (e.g., in-situ and geotechnical investigations), yet complex engineering scenarios often cannot be fully resolved experimentally. Therefore, numerical simulations should complement experimental analyses where feasible, validating and supplementing field data to provide actionable guidance for on-site testing. To simulate liquefaction resistance of gravel pile-reinforced foundations under parametric variations, researchers have employed diverse three-dimensional finite-difference software. Jiang et al. [12] numerically analyzed deformation in expressway gravel pile composite foundations under varying conditions: pile construction processes, layered embankment/pavement filling, and traffic loads. Using FEMEPDYN, Li [13] proved that arranging gravel piles with long-short alternation (long piles on sides, short in center) enhances both liquefaction resistance and cost-efficiency in sand foundations. Huang [14] systematically reviewed prior studies and concluded that gravel piles provide comparable liquefaction resistance in silty and sandy foundations. Zhang [15] compared concrete piles versus gravel piles for reinforcing inclined liquefiable sites, finding concrete piles superior under identical embedment depths and pile diameters. Through numerical simulation, Shen [16] demonstrated that extending gravel pile lengths in saturated silt layers significantly reduces vertical/horizontal displacements of subway shield tunnels. For the Sihu Expressway’s silty sand foundation, Wang [17] implemented gridded gravel piles. Experimental tests and FLAC^3D^ analyses demonstrated their dual role: drainage/pressure relief for liquefaction mitigation and enhanced shear capacity, proving highly effective against seismic liquefaction in saturated silt. Gu [18] investigated gravel piles as anti-liquefaction measures for bridge foundations, detailing design methodologies and validation approaches. Niu et al. [19] developed a full soil-structure model in FLAC^3D^ to quantitatively assess soil-cement pile efficacy via residual deformation, excess pore pressure (ratio), and surface acceleration. Liu [20] simulated single versus group gravel piles reinforcing liquefiable sandy foundations via FEM, revealing superior performance of pile groups. Using FLAC^3D^’s Finn module, Li [21] confirmed that gravel piles significantly improve dynamic responses in both structures and soil layers. Qiu [22] proposed and validated an OpenSees-based evaluation framework for assessing liquefaction resistance in gravel pile-reinforced saturated sand foundations, corroborated by model tests. Through three-dimensional finite element simulations in OpenSeesPL, Asgari et al. [23] parametrically analyzed gravel piles and dowel pins for liquefaction mitigation. Key factors included area replacement ratio, soil/pile permeability, slope angle, pile diameter, superstructure mass, and seismic characteristics.

In this study, we establish dynamic analysis models of composite foundations for the saturated sand foundation of a marine diversion dike at a coastal nuclear power plant, comparing pre- and post-gravel pile construction conditions using FLAC^3D^ (Fast Lagrangian Analysis of Continua). Employing the PL-Finn constitutive model [24] for post-liquefaction large deformation, we conduct dynamic analyses to evaluate the anti-liquefaction efficacy of compacted gravel piles through three key indicators: residual deformation, excess pore water pressure, and pore pressure ratio. This approach enables comprehensive assessment of the anti-liquefaction properties, mechanisms, and reinforcement effects of gravel pile composite foundations, establishing a basis for optimizing gravel pile foundation reinforcement schemes.

## 2 Principles of calculation and analysis

### 2.1 Principle of FLAC^3D^ dynamic analysis

FLAC^3D^ adopts a completely nonlinear dynamic response analysis method, which can accurately simulate plastic collapse and plastic flow characteristics of geomaterials. Besides, in the dynamic analysis module, the user-defined nonlinear constitutive model can be used to reflect the stress-strain hysteresis and deformation characteristics of soil. In addition, FLAC^3D^ can also adopt a fully coupled fluid-mechanics analysis model, in which the pore pressure response of the element affects the plastic volume deformation, and the plastic volume deformation of the element affects the pore water pressure. It can simulate the law of pore pressure growth and dissipation during the sand liquefaction process. The basic controlling formula of liquid-solid coupling in FLAC^3D^ dynamic calculation is described by the following fluid continuity formula:


$$\frac{1}{M}\frac{\partial p}{\partial t}=\frac{\partial \zeta }{\partial t}-\alpha \frac{\partial \varepsilon }{\partial t}$$ (1)

In the above formula, ζ is the impulse boundary, which is defined as the volume change per unit volume of fluid in porous media. ε represents body strain; the calculation size is related to the elastoplastic constitutive formula. *M* is the Biot modulus and α is the Biot coefficient.

### 2.2 Liquefaction discrimination

Under dynamic action such as earthquakes, the saturated foundation will be changed from solid to liquid during liquefaction. When the viscous force of liquid is not considered, its shear strength will be changed to 0. In other words, when effective principal stress is 0, the saturated foundation will be liquefied. According to the principle of effective stress, the principal stress in three directions of the soil should be equal to the pore water pressure. Therefore, $\sigma_{1}=\sigma_{2}=\sigma_{3}=\mu$.${\;\sigma }_{i}(i=1,2,3)$ is the principal stress in three directions of soil during liquefaction, kpa; μ is the pore water pressure during liquefaction, kpa.

The excess pore water pressure ratio $r_{u}\;$ can be expressed as:


$$r_{u}=1-\frac{\sigma_{m}^{\prime }}{\sigma_{m0}^{\prime }}$$ (2)

$$\begin{array}{c} \sigma_{m0}^{\prime }=\frac{\left(\sigma_{10}^{\prime }+\sigma_{20}^{\prime }+\sigma_{30}^{\prime }\right)}{3} \end{array}$$ (3)

$$\begin{array}{c} \sigma_{m}^{\prime }=\frac{\left(\sigma_{1}^{\prime }+\sigma_{2}^{\prime }+\sigma_{3}^{\prime }\right)}{3} \end{array}$$ (4)

$\;\sigma_{m0}^{\prime }\;$ is the average effective stress of the unit before the dynamic calculation, kpa and $\sigma_{m}^{\prime }\;$ is the average effective stress of the unit during the dynamic calculation, kpa.

### 2.3 Fluid constitutive model after liquefaction

The constitutive model of sand flow characteristics after liquefaction is mainly established according to two different states of sand, namely zero effective stress state and non-zero effective stress state. The shear stress-shear strain rate relationship under zero effective stress state is as follows:


$$\begin{array}{c} q_{0}=k_{0}{\left({\dot{\varepsilon }}_{0}\right)}^{n_{0}} \end{array}$$ (5)

The shear stress-shear strain rate relationship under a non-zero effective stress state is:


$$\begin{array}{c} \log\left(\frac{q_{i}}{{\dot{\varepsilon }}_{i}}\right)=k_{i}{\left(1-r_{u}\right)}^{n_{i}} \end{array}$$ (6)

In this formula, $q_{i}$ represents the generalized shear stress, kpa. ${\dot{\varepsilon }}_{i}$ represents the generalized strain rate; $k_{i}$, $n_{i}$ are the fitting parameters, and $r_{u}$ is the excess pore water pressure ratio.

The Finn model takes deformation reversal into account and analyzes the problem in three dimensions, with at least six strain rate tensor components. These six strain rate tensors are defined as:


$$\varepsilon_{1}:=\varepsilon_{1}+\Delta e_{12};\varepsilon_{2}:=\varepsilon_{2}+\Delta e_{23};\varepsilon_{3}:=\varepsilon_{3}+\Delta e_{31};$$

$$\begin{array}{c} \varepsilon_{4}:=\varepsilon_{4}+\frac{\left(\Delta e_{11}-\Delta e_{22}\right)}{\sqrt{6}};\varepsilon_{5}:=\varepsilon_{5}+\frac{\left(\Delta e_{22}-\Delta e_{33}\right)}{\sqrt{6}};\varepsilon_{6}:=\varepsilon_{6}+\frac{\left(\Delta e_{33}-\Delta e_{11}\right)}{\sqrt{6}} \end{array}$$ (7)

In this formula, e_ij_ is the strain increment tensor.

The extreme point of its trajectory is found in the strain space. (^o^) represents the previous point, (^oo^) represents the second point, and the previous unit vector n^o^ in the strain space is calculated as follows:


$$\begin{array}{c} v_{i}=\varepsilon_{i}^{0}-\varepsilon_{i}^{00} \end{array}$$ (8)

$$\begin{array}{c} n_{i}^{o}=\frac{V_{i}}{\sqrt{V_{i}V_{i}}} \end{array}$$ (9)

In this formula, v_i_ represents the strain increment on each component, and the subscript i represents 1–6 components.

New $\varepsilon_{i}-\varepsilon_{i}^{0}$ projection d onto the previous unit vector d is:


$$\begin{array}{c} d=\left(\varepsilon_{i}-\varepsilon_{i}^{0}\right)n_{i}^{0} \end{array}$$ (10)

If d < 0, the strain is reversed. The absolute value of d is monitored during calculation. When the minimum calculation period is reached, if |d| reaches the maximum value |d|>=d _max_, then the inverse calculation is carried out, that is:


$$\begin{array}{c} \gamma =d_{\max}{;\varepsilon }_{i}^{00}{=\varepsilon }_{i}^{0};\varepsilon_{i}^{0}{=\varepsilon }_{i} \end{array}$$ (11)

After shear strain γ and plastic volume strain increment $\Delta \varepsilon_{vd}$ are obtained, the plastic volumetric strain is updated:


$$\begin{array}{c} \varepsilon_{vd}:=\varepsilon_{vd}+\Delta \varepsilon_{vd} \end{array}$$ (12)

According to $\Delta \varepsilon_{vd}$, the shear strain increment can be modified:


$$\begin{array}{c} \Delta e_{ij}:=\Delta e_{ij}+\frac{\Delta \varepsilon_{vd}}{3} \end{array}$$ (13)

In FLAC^3D^, the compressive strain increment has negative values and the plastic volume strain has positive values. Therefore, the average effective stress gradually decreases with the increase of plastic volume strain accumulation.

Based on the original liquefaction calculation model (the Finn model), the professional software of geotechnical engineering FLAC^3D^ has improved and added the post-liquefaction flow calculation module to form the PL-Finn model. It can more comprehensively consider the initial consolidation state and stress-strain relationship of soil before liquefaction, as well as the residual stress and residual deformation of soil after liquefaction. The PL-Finn model development procedure diagram is illustrated in S1 Fig.

### 2.4 Liquefaction mechanism of foundation elimination by gravel piles

The principle of vibration gravel piles to reinforce soft soil foundation is mainly to replace part of soft soil, form holes in sand, silty soils, cohesive soil, artificial fill, miry soil and other soil layers, and then backfill gravel and other coarse materials to form pile. Moreover, form a composite foundation with the original foundation soil to meet the bearing capacity required by the project, meet the settlement less than the limit value of the project, reduce deformation and compressibility and eliminate soil liquefaction. The principle of anti-liquefaction of gravel piles is mainly divided into three aspects: (1) compacting effect; (2) drainage effect; (3) pre-vibration effect. The bearing capacity of the gravel pile composite foundation is composed of the bearing capacity characteristic value f_spk_ of the gravel piles in the foundations and the bearing capacity characteristic value f_sk_ of soil between piles.

## 3 Engineering cases

### 3.1 Engineering situation

**Fig 1:**
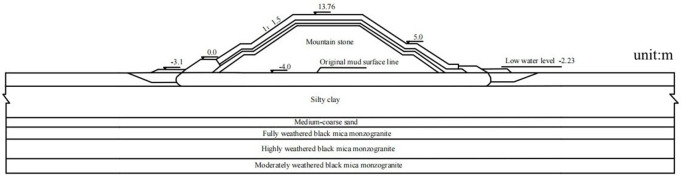
Diagram of diversion dike structure and foundation cross-section.

The marine engineering infrastructure for a nuclear power plant primarily comprises diversion dikes and revetments. This study focuses on a typical cross-section of the diversion dike. The site’s upper strata consist of Quaternary deposits (marine, alluvial, and residual), underlain by Mesozoic bedrock. From top to bottom, the foundation soils include: mucky soil, silty clay, medium-coarse sand, fully weathered granite, highly weathered granite, and moderately weathered granite. Based on local hydrogeological data, the design water level is set at −2.23 m below datum. Fig 1 illustrates the diversion dike structure and foundation cross-section.

### 3.2 Calculation models and parameters

**Fig 2:**
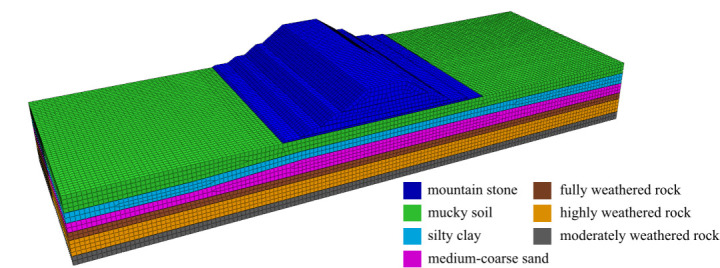
Three-dimensional finite element model of diversion dike.

Fig 2 shows the three-dimensional finite element model of the diversion dike. The model is 300m long, 100m wide and 55m high. It is composed of 134,242 units and 144,017 nodes. The unit size is 1.5-2.5m. In the analysis, the medium-coarse sand layer between silty clay and fully weathered rock is set as the liquefaction layer; the PL-Finn model is used for medium-coarse sand layer, and the Mohr-Coulomb model is used for other soil layers.

**Fig 3:**
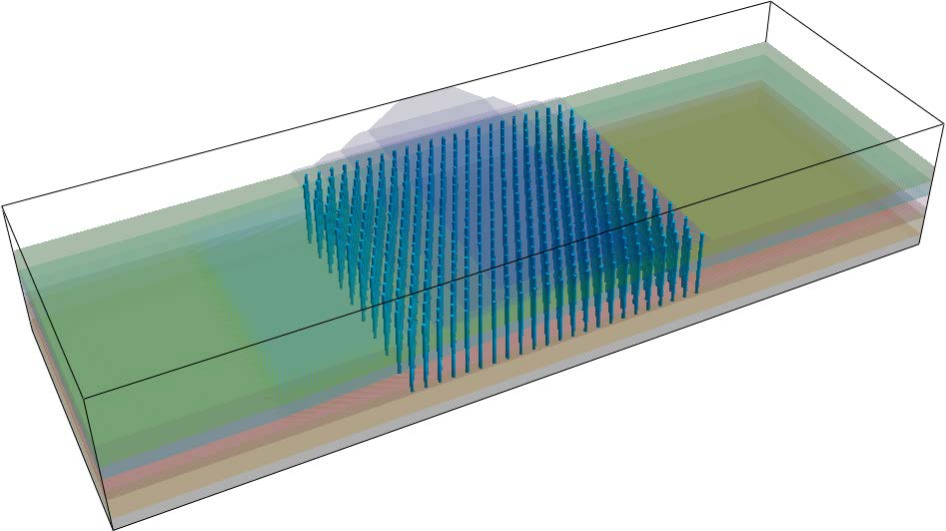
Three-dimensional finite element model of the diversion dike after reinforcement.

**Fig 4:**
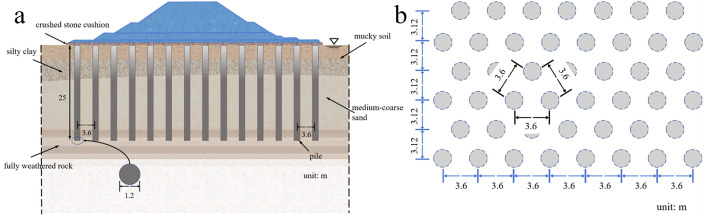
Schematic diagram of gravel pile group arrangement (a) Plan view schematic (b) Pile layout schematic.

Fig 3 is a three-dimensional finite element model of the diversion dike after reinforcement. The pile structural unit of FLAC^3D^ defines the compacted gravel pile group. Several scholars analyzed the influence range of pile diameter, pile length and pile spacing on pore water pressure, compaction degree and soil around piles by means of model tests and numerical simulation, and they proposed the reasonable pile spacing should be 2.5d ~ 3d [25]. Therefore, the foundation reinforcement adopts an equilateral triangle pile layout, in which the gravel pile diameter is 1.2 m, gravel pile spacing is 3.6m, and 450 piles in total. The length of the gravel pile is set at 25m to penetrate the mucky layer, silty clay layer and medium-coarse sand layer. A gravel cushion layer with 0.5m thickness is laid between the top of the compacted gravel pile and the diversion dike. Fig 4 shows the layout plan of the gravel pile.

The mechanical parameters of rock-soil mass, fluid parameters and liquefaction parameters in the analysis are shown in Tables 1–3. The parameters are selected according to the *Geotechnical Engineering Investigation Report* and *Test Report on Static* and *Dynamic Characteristics of Rock and Soil* of the nuclear power plant. The soil mechanics parameters and contact surface parameters of the compacted gravel piles are shown in Table 4 and 5 below. The research direction of the contact surface is the “soft” contact surface. Nuclear power engineering adopts a conservative design, and it is necessary to consider the ultimate load-bearing condition when the contact surface is in a completely smooth state. At this time, the anti-slip ability of the structure is the weakest. Thus, specific friction angle parameters are assigned to the contact surface.

**Table 1:** Residual deformation parameters of soil.

Materials	Dynamic shear modulus coefficient *K*	Index *n*	*C*_1_	*C*_2_	*C*_3_	*C*_4_	*C*_5_
**Mucky soil**	76.8	0.76	0.006206	1.0691	0	0.9671	1.1869
**Silty clay**	421.1	0.84	0.000608	0.7403	0	0.8745	1.7854
**Medium-coarse sand**	849.1	0.45	0.000530	0.9357	0	0.2599	1.4481
**Fully weathered rock**	527.4	0.50	0.006868	0.9898	0	3.0196	1.1747

**Table 2:** Soil mechanical parameters.

Materials	Saturation capacity γ/ (kN•m^-3^)	Elastic modulus E/ kPa	Cohesion *c*/ kPa	Internal friction angle *φ*/°	Poisson ratio *μ*
**Mountain stone**	17.0	500000	0.0	42.0	0.33
**Mucky soil**	15.7	4500	10.3	13.0	0.38
**Silty clay**	19.8	30000	29.1	12.0	0.35
**Medium-coarse sand**	20.0	28200	26.1	21.8	0.33
**Fully weathered rock**	18.2	15000	18.5	17.3	0.33
**Highly weathered rock**	20.0	390000	40.0	40.0	0.33
**Bedrock**	26.3	1200000	200.0	40.0	0.30

**Table 3:** Soil fluid and liquefaction parameters.

Materials	Permeability coefficient/ (m^2^/Pa-sec)	Porosity *n*	Damping ratio	Liquefaction parameters (only medium-coarse sand)	
**Mountain stone**	1.0E-08	0.48	0.05	C_1_ = 0.80 C_2_ = 0.79 C_3_ = 0.45 C_4_ = 0.73	pprc = 0.99 *k*_*0*_ = 3105.4 *n*_*0*_ = 0.3225 *k*_*1*_ = 5503.1 *n*_*1*_ = 0.1739
**Mucky soil**	1.0E-13	0.45	0.05		
**Silty clay**	1.0E-12	0.44	0.05		
**Medium-coarse sand**	1.0E-09	0.46	0.05		
**Fully weathered rock**	1.0E-12	0.43	0.05		
**Highly weathered rock**	1.0E-13	0.42	0.05		
**Moderately weathered rock**	–	–	0.05		

**Table 4:** Soil mechanical parameters of compacted gravel piles.

Materials	Dry density ρd/ (kg/m3)	Elastic modulus E/MPa	Cohesion c/kPa	Internal friction angle *φ*/°	Poisson ratio μ
Plie	2500	30000	–	–	0.3

**Table 5:** Parameters of the contact surface.

Normal stiffness *K*_*n*/_(Pa/m)	Normal stiffness *K*_*s/*_(Pa/m)	Cohesion *C*_*if/*_kPa	Friction angle *φ*_*if*_/°
1.2 × 10^8^	1.2 × 10^8^	21	0

The bulk modulus and shear modulus are required for calculation and analysis in FLAC^3D^, but the above parameters are not provided. Therefore, calculate according to the following formula:


$$\begin{array}{c} K=\frac{E}{3(1-2v)} \end{array}$$ (14)

$$\begin{array}{c} G=\frac{E}{2(1+\nu )} \end{array}$$ (15)

In this formula, K is the bulk modulus, G is the shear modulus, E is the Yang’s modulus, ν is the Poisson ratio.

When doing fluid calculations in FLAC^3D^, the permeability coefficient K is one of the key parameters. Usually, the permeability coefficient K(cm/s) of soil mechanics provided in the investigation report cannot be directly applied to FLAC^3D^. It needs to be converted by the formula in the calculation.


$$\begin{array}{c} k\left(\frac{m^{2}}{Pa}-sec\right)=K\left(\frac{cm}{s}\right)\times 1.02\times 10^{-6} \end{array}$$ (16)

### 3.3 The setting of seismic dynamic loads and dynamic boundary conditions

**Fig 5:**
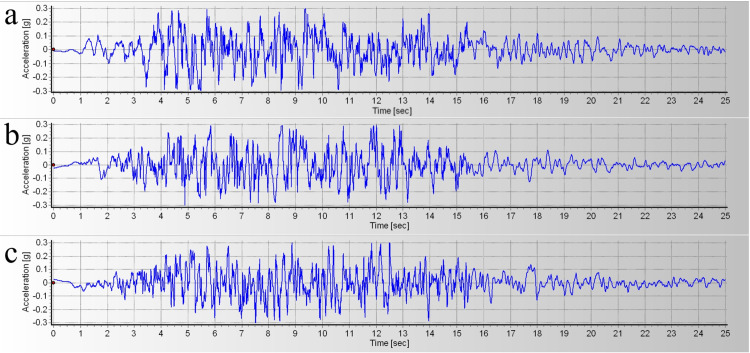
Seismic wave time-history curve (a) horizontal direction 1 (b) horizontal direction 2 (c) vertical direction.

**Fig 6:**
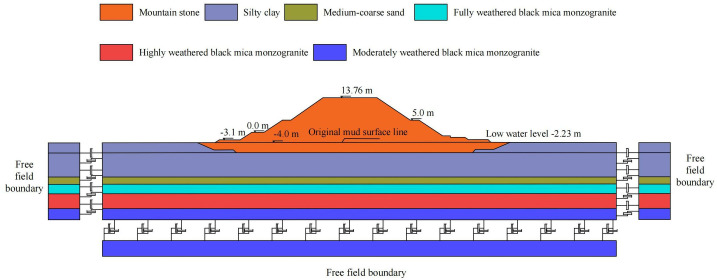
The schematic diagram of free-field boundary.

According to the seismic safety assessment materials of a coastal nuclear power plant, the dynamic time-history analysis adopts the improved RG1.60 response spectrum of the United States, whose damping ratio is 5% and the duration is 25s. The corresponding time-history curve of seismic waves is shown in Fig 5. The design value of the horizontal peak acceleration of the bedrock is 0.30g, and the vertical peak acceleration is 0.20g. The free field boundary is set around the dynamic calculation model, and local damping is set to make the finite element mesh of the main structure coupled with the free field mesh, and the damping coefficient is 0.157. The boundary condition schematic diagram is shown in Fig 6.

### 3.4 Basic assumptions of the model

The numerical simulation analysis of the model calculation process conforms to the following four assumptions [26]: (1) the soil in numerical models is saturated; the water in the soil seepage process conforms to the Darcy law; (2) soil grain and pore water is incompressible; (3) set the material property of the gravel piles as isotropic linear elasticity; (4) considering the numerical stability of the PL-Finn model, the compression coefficient and permeability coefficient of the soil are set as constant, and the permeability coefficients are equal in each direction.

### 3.5 The setting of monitoring points

**Fig 7:**
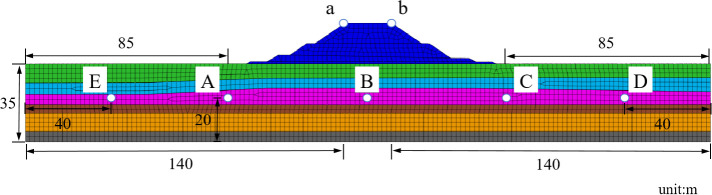
Schematic diagram of monitoring points and units.

To monitor structural displacement and the excess pore water pressure ratio in the medium-coarse sand layer, monitoring points are installed on both sides of the diversion dike top, while monitoring units for this ratio are embedded within the sand layer. Fig 7 is the Schematic diagram of monitoring points and units. Table 6 is model monitoring points and element coordinates.

**Table 6:** Model monitoring points and units coordinates.

Monitoring points	X/(m)	Y/(m)	Z/(m)
**A**	85	50	20
**B**	150	50	20
**C**	215	50	20
**D**	260	50	20
**E**	40	50	20
**a**	140	50	55
**b**	160	50	55

### 3.6 Analysis procedure

**Fig 8:**
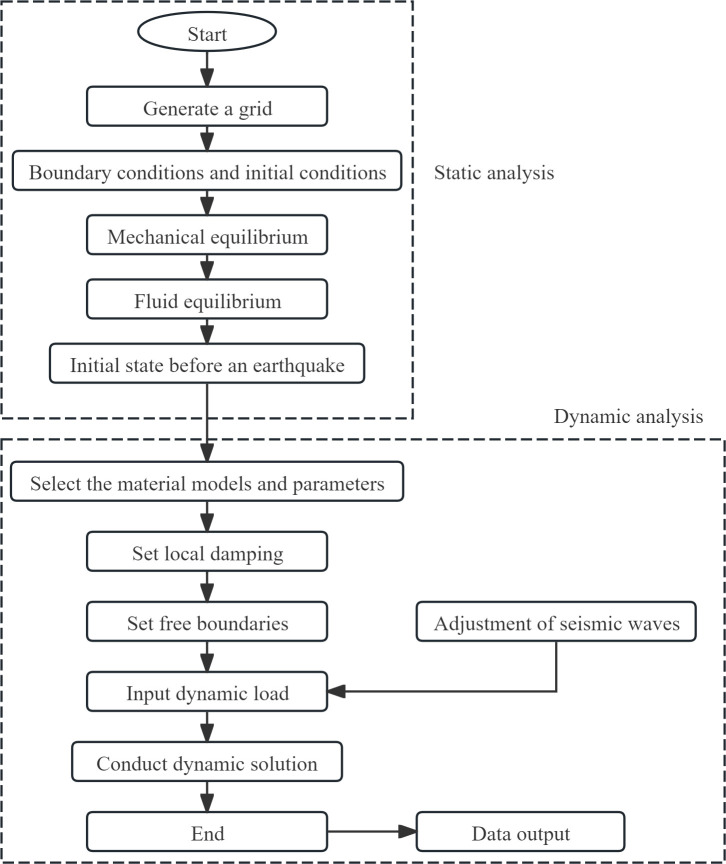
The analysis procedure.

When simulating the dynamic response of a composite foundation under earthquake action, static analysis and seepage flow calculation of the model should be conducted first to obtain the correct initial ground stress field and seepage field. During dynamic calculation, issues such as material model and parameters, boundary conditions, and damping types should be taken into consideration. The specific analysis procedure flowchart is shown in Fig 8.

## 4 Analysis of numerical simulation results

### 4.1 Analysis of the residual deformation

**Fig 9:**
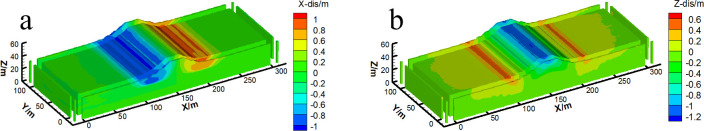
Cloud chart of residual deformation distribution before reinforcement (a) Cloud chart of horizontal residual deformation distribution (b) Cloud chart of Vertical residual deformation distribution.

**Fig 10:**
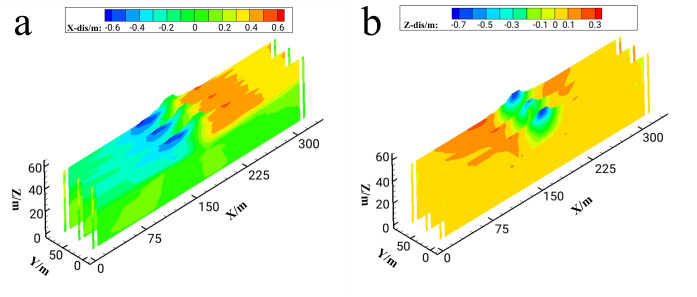
Cloud chart of residual deformation distribution after reinforcement (a) Cloud chart of horizontal residual deformation distribution (b) Cloud chart of Vertical residual deformation distribution.

**Fig 11:**
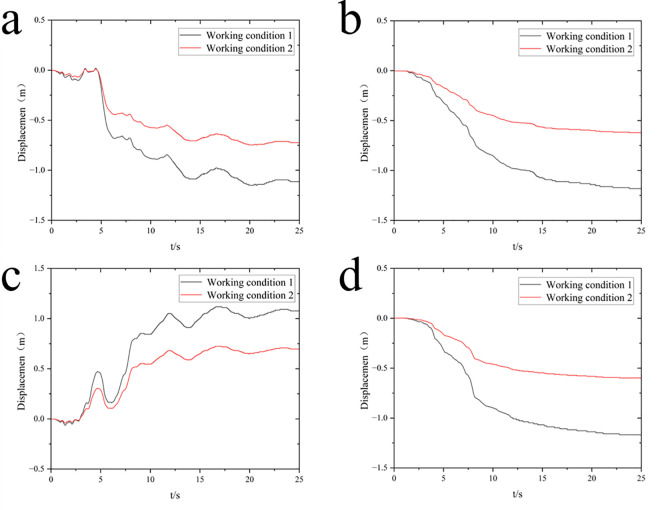
Time-history curve of deformation at monitoring points before and after reinforcement (a) Horizontal displacement deformation curve of point a (b) Vertical displacement deformation curve of point a (c) Horizontal displacement deformation curve of point b (d) Vertical displacement deformation curve of point b.

The residual deformation cloud charts for the conditions before and after gravel pile reinforcement are shown in Figs 9 and 10, with deformation time-history curves at monitoring points shown in Fig 11. Comparative analysis of residual deformation under the two working conditions indicates that liquefaction in bilateral sand layers induces symmetrical lateral displacement of the diversion dike about its central axis, as evidenced by the horizontal residual deformation cloud chart. In contrast, the underlying sand layer exhibits minimal displacement due to the absence of liquefaction, while maximum horizontal displacement occurs at both sides of the structure.

According to the time-history curve of horizontal displacement deformation of the monitoring points, the horizontal displacement of monitoring point a is 1.11m and that of monitoring point b is 1.0m before reinforcement. After reinforcement, the horizontal displacement of monitoring point a is 0.72m, and monitoring point b is 0.69m. The residual deformation of horizontal displacement is reduced by 35%.

From the vertical residual deformation distribution cloud chart, the whole diversion dike has uniform settlement under earthquakes, and the largest settlement occurs at the top of the diversion dike. The foundation settlement range below the structure gradually decreases with the increase of depth, and the heave deformation occurs on the contact position between the two sides of the structure and the mucky soil. It can be found that from the time-history curve of vertical displacement deformation, before reinforcement, the subsidence of the monitoring points a and b is 1.18m and 1.17m respectively. After reinforcement, the subsidence is reduced to 0.62m and 0.60m. The residual deformation of vertical settlement is reduced by 47%. According to the reduction of vertical and horizontal residual deformation of the diversion dike, it can be proposed that the seismic performance of the diversion dike has been significantly improved.

### 4.2 Analysis of the excess pore water pressure

When saturated sand is subjected to external loads, its volume decreases as the particles are compressed. This compression causes the pore water pressure to increase steadily, eventually exceeding the original hydrostatic pressure. The pressure in excess of the hydrostatic pressure is termed the excess pore water pressure. The development of excess pore water pressure is a key indicator of the liquefaction state in saturated sand.

**Fig 12:**
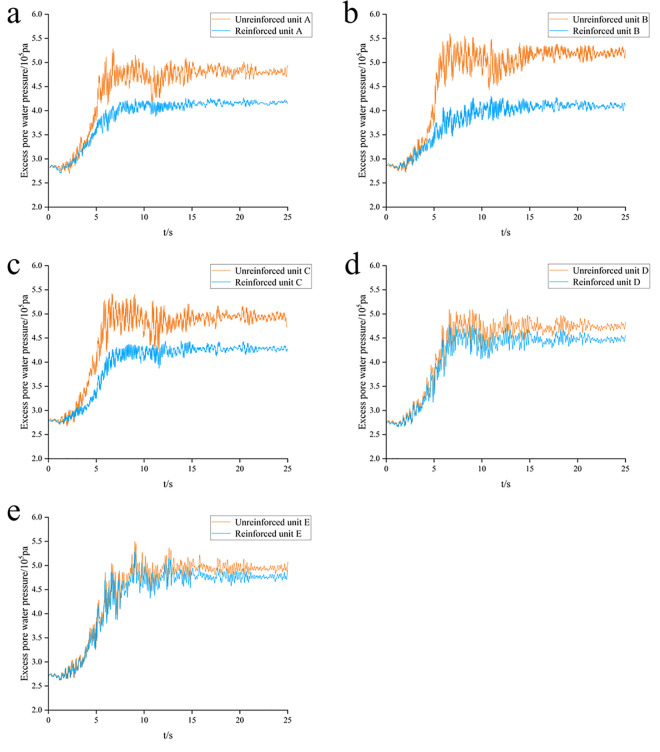
Time-history curves of excess pore water pressure for different monitoring units: (a) Element A (b) Element B (c) Element C (d) Element D (e) Element E.

Fig 12 shows the time-history curves of excess pore water pressure for each monitoring unit before and after reinforcement. Following the application of seismic waves at the model base, the excess pore water pressure at each unit rapidly peaks, then decreases slightly, and finally stabilizes. As the distance from the pile center increases, the excess pore water pressure rises, indicating a progressive weakening of the gravel piles’ drainage effect. Comparison shows that peak excess pore water pressures in the reinforced area are significantly lower after reinforcement, whereas peaks in the unreinforced area show little change. These results demonstrate that compacted gravel piles provide effective drainage, facilitating the dissipation of excess pore water pressure in the medium-coarse sand layer and thereby reducing liquefaction potential.

### 4.3 Analysis of the excess pore water pressure ratio

In this study, FLAC^3D^ performs numerical simulations and analyses.The excess pore water pressure ratio, defined as the ratio of pore water pressure increment to lateral effective consolidation stress, serves as the liquefaction criterion to ensure computational accuracy. Experimental data [27] indicate that liquefaction initiation occurs when this ratio reaches 0.87 in silty soils, with complete liquefaction at 1.0. Herein, a threshold ratio of 0.8 is adopted to define the liquefaction state, implemented through FISH programming for concurrent computation.

**Fig 13:**
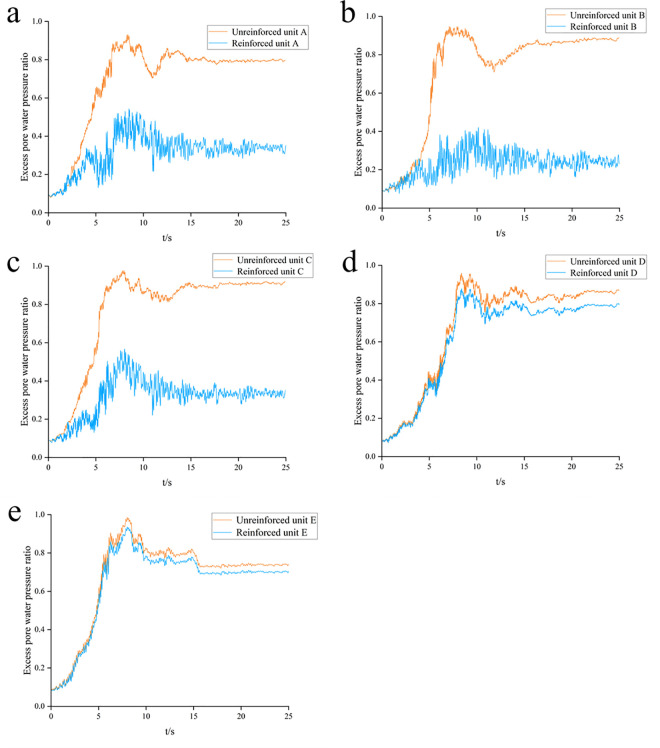
Time-history curves of excess pore pressure ratio for different monitoring units: (a) Element A; (b) Element B; (c) Element C; (d) Element D; (e) Element E.

**Fig 14:**
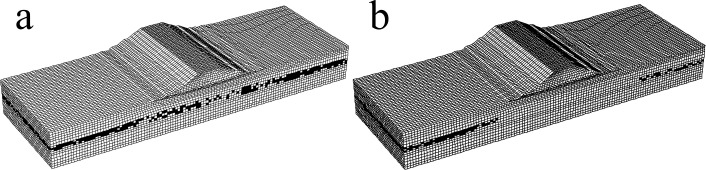
Distribution of liquefied zones in the three-dimensional model: (a) Before reinforcement; (b) After reinforcement.

Fig 13 compares the time-history curves of the excess pore water pressure ratio for each monitoring point before and after reinforcement. The results show that installing compacted gravel piles in the saturated sand foundation significantly reduces the peak excess pore water pressure ratio at monitoring points within the reinforced area, but causes little change outside it. Before reinforcement, the excess pore water pressure ratio rises rapidly at 7s and then falls rapidly, stabilizing at 14s. This indicates that sand liquefaction occurs (when effective stress reaches zero), followed by gradual dissipation of pore water pressure. During seismic loading, the excess pore water pressure ratio increases with increasing horizontal distance from the pile center, indicating a progressive weakening of the gravel piles’ drainage effect. Consequently, the anti-liquefaction performance of the gravel pile-reinforced composite foundation is significantly enhanced. Within the reinforced area, the peak excess pore water pressure ratio near the center is about 0.4, while near the edge it ranges between 0.45 and 0.5. Outside the reinforced area, the sand layer experiences varying degrees of liquefaction. Therefore, the goal of eliminating site liquefaction is largely achieved under the design-level earthquake intensity. Fig 14 shows the distribution of liquefied zones in the three-dimensional model for both (pre- and post-reinforcement) conditions.

## 5 Discussion

In this study, we adopt the PL-Finn constitutive model—a secondary development of the Finn model incorporating post-liquefaction flow deformation behavior. Numerical simulations of the diversion dike closely align with field investigation data. Compared to the original Finn model, the PL-Finn model more accurately captures deformation patterns and liquefaction zones in saturated sand under dynamic loading. All conventional mechanical parameters required for static and dynamic analyses can be determined through laboratory tests or empirical formulas, granting the model strong practical applicability in engineering.

Observations (Figs 9–10) reveal that post-liquefaction sand flow induces regular residual deformation: the structure undergoes near-symmetrical lateral sliding on both flanks and uniform upper-section settlement, with magnitude decreasing progressively with depth. Gravel pile reinforcement triggers lateral heave deformation in liquefiable soils outside the reinforced zone due to foundation subsidence-induced compression, exhibiting asymmetric patterns. These characteristics align with Zou’s findings [28]. Mechanistically, adjacent unreinforced soils exhibit limited drainage capacity, causing delayed dissipation of excess pore pressure post-liquefaction. Consequently, low volumetric strain and weak deformation resistance lead to significant lateral and upward displacement when compressed by overlying foundations.

Notably, Adampiro and Derakhshandi [29] investigated layered liquefiable soils’ effects on seismic site response via experiments and numerical simulations. Their findings clarify that the relative thickness of the liquefiable layer and overlying non-liquefied crust govern seismic wave attenuation, amplification, and ground motion intensity, consequently altering liquefaction characteristics. Fig 12 reveals higher peak excess pore pressure at the center versus sides, attributed to greater sand layer thickness—validating the thickness-liquefaction correlation. Liquefaction initiates when the excess pore pressure ratio exceeds 0.8 in sand layers under seismic loading (Fig 13). Given the unique requirements of nuclear power plant marine structures and the diversion dike’s critical role in operational safety, foundation reinforcement becomes paramount.

Additionally, Iranian scholars [30] demonstrated that gravel piles enhance drainage within about 2.5m from their center, with effects diminishing beyond this radius—corroborating our findings on effective liquefaction mitigation ranges. Their study recommends optimal settlement control at pile spacings of 2.5–3.5 times pile diameter [30], aligning with our design (Fig 4). Notably, El Sherbiny and Wang [31] determined square patterns outperform triangular or hexagonal arrangements for tunnel liquefaction mitigation. Closer pile spacing enhances soil stiffness and enables uniform drainage, thereby reducing excess pore pressure and tunnel uplift. While distinct from our focus, future work should assess pile arrangements (spacing, diameter, pattern) to optimize cost-effective liquefaction countermeasures.

Owing to the numerical stability constraints of the PL-Finn model, this study adopted the assumption of constant soil permeability. However, pore structure rearrangement during liquefaction may alter permeability, thereby affecting the dissipation of excess pore water pressure and the drainage efficiency of gravel piles. To address this knowledge gap, we will quantify the effects of permeability variations on pore water pressure dissipation and drainage efficiency in ongoing research (S1 File).

In this study, due to the conservative design philosophy inherent in nuclear power engineering, the pile-soil contact was modeled under ultimate bearing conditions assuming a perfectly smooth state with zero friction angle. This setting may lead to an underestimation of the lateral load transfer efficiency of stone columns, while potentially overestimating foundation settlement to some extent, resulting in conservative simulation outcomes. This approach aligns with the principle of prioritizing computational efficiency. Although the results obtained from the numerical simulations demonstrate significant potential for application in the design of nuclear marine infrastructure, these conclusions have not yet been validated through field data or benchmark testing due to current research limitations. To enhance the reliability and generalizability of the findings, we will conduct scaled shaking table model tests on diversion dikes in future research. Systematic comparison between measured dynamic responses and three-dimensional numerical simulation results will validate the rationality of constitutive model parameter selection and the effectiveness of the computational methodology.

## 6 Conclusion

Based on the actual liquefaction engineering of a coastal nuclear power project, this study studies the dynamic characteristics and anti-liquefaction reinforcement effect of the composite foundation of gravel piles in liquefiable sites, and carries out numerical analyses of the seismic dynamic response of the composite foundation-foundation-superstructure system of gravel piles in liquefiable sites. The main conclusions are as follows:

(1) Without reinforcement, liquefaction occurs; gravel pile reinforcement significantly enhances liquefaction resistance. In the reinforced zone, the maximum excess pore pressure ratio at the foundation base is about 0.50, preventing liquefaction under design-level ground motion.
(2) During the earthquake, the excess pore water pressure at each monitoring point rapidly increases to the peak value, then decreases slightly, and finally hovers around the stable value. Gravel pile reinforcement significantly reduces the peak excess pore water pressure in the composite foundation, demonstrating that gravel piles can effectively dissipate excess pore water pressure and promptly discharge excess pore water.
(3) While improving the liquefaction resistance of the reinforced area, gravel pile reinforcement greatly reduces the seismic settlement and displacement of liquefiable sites. Before reinforcement, the maximum lateral slip is about 1.11 m, and the maximum vertical settlement is about 1.18m. After reinforcement, the maximum lateral slip is about 0.72m, and the maximum vertical settlement is about 0.62 m. The vertical and horizontal residual deformation of the diversion dike is significantly reduced, and the stability of the structure is greatly improved.

## Supporting information

S1 File PL-Finn Model-based computational code for liquefaction analysis. (ZIP)

S1 Fig PL-Finn model development procedure diagram. (TIF)
