# Supplementary figures and images for: Liquefaction analysis of marine diversion dike foundation and gravel pile reinforcement treatment based on the PL-Finn model
Source: PLoS One. 2025 Aug 12;20(8):e0330325. doi: 10.1371/journal.pone.0330325 (PMC12342268; doi:10.1371/journal.pone.0330325)

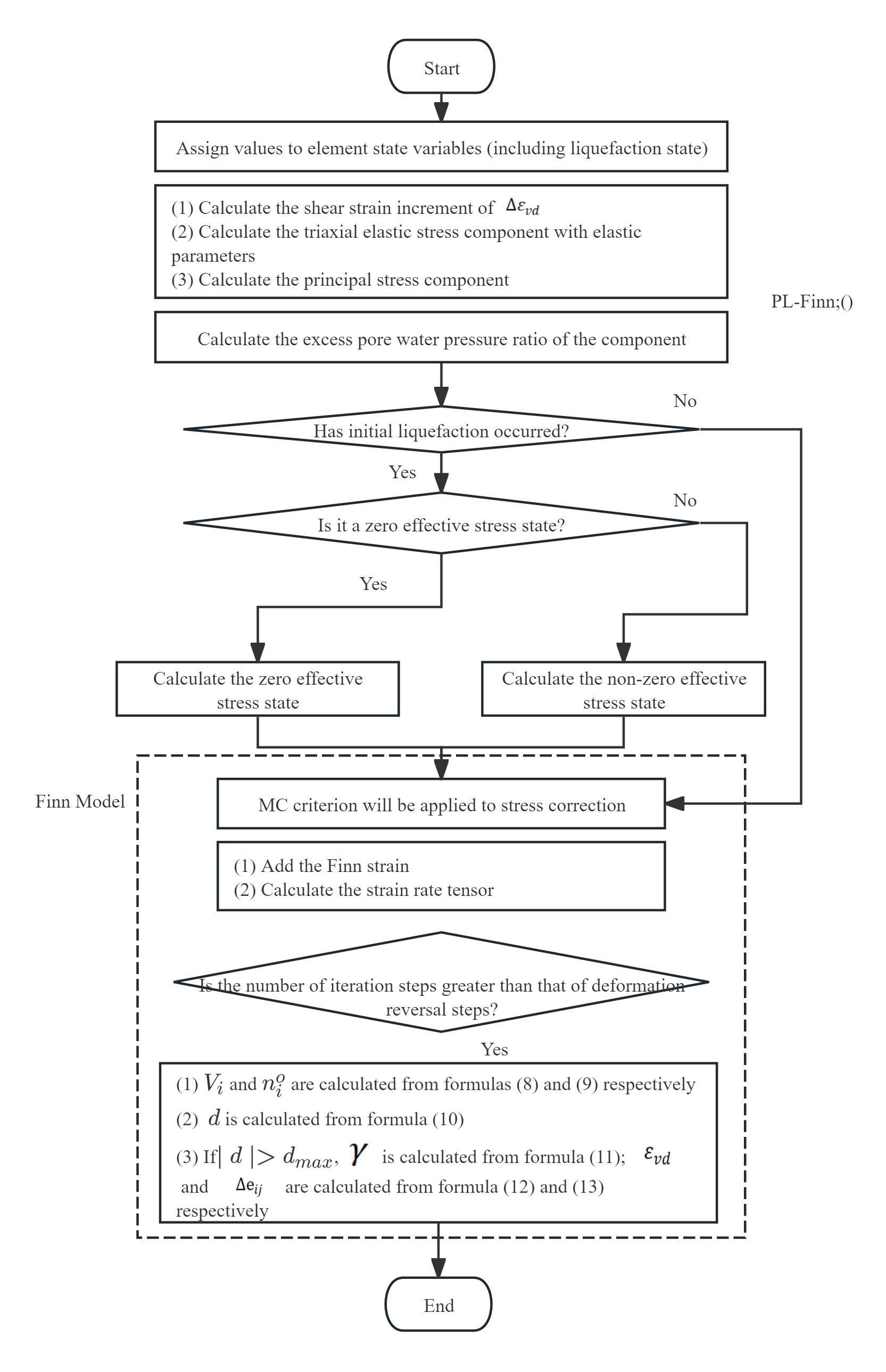

Supplement: S1 Fig — (TIF) [file pone.0330325.s002.tif]
